# Investigating Emotion in Malay, Australian and Iranian Individuals with and without Depression

**DOI:** 10.1038/s41598-019-54775-x

**Published:** 2019-12-04

**Authors:** Laura Jobson, Vida Mirabolfathi, Shiva Moshirpanahi, Hadi Parhoon, Julia Gillard, Firdaus Mukhtar, Ali Reza Moradi, Sindhu Nair Mohan

**Affiliations:** 1 Turner Institute for Brain and Mental Health, Monash University, Melbourne, Australia; 2 Kharazmi University and Institute for Cognitive Science Studies, Tehran, Iran; 3 Razi University, Kermanshah, Iran; 4 University College London, London, UK; 5 Universiti Putra Malaysia, Selangor, Malaysia

**Keywords:** Human behaviour, Diseases

## Abstract

This study investigated the influence of culture and depression on (1) emotion priming reactions, (2) the recall of subjective experience of emotion, and (3) emotion meaning. Members of individualistic culture (Australia, *n* = 42) and collectivistic culture (Iran, *n* = 32, Malaysia, *n* = 74) with and without depression completed a biological motion task, subjective experience questionnaire and emotion meaning questionnaire. Those with depression, regardless of cultural group, provided significantly fewer correct responses on the biological motion task than the control group. Second, the collectivistic control groups reported greater social engaging emotion than the Australian control group. However, the three depressed groups did not differ culturally. The Australian depressed group reported significantly greater interpersonally engaging emotion than the Australian control group. Third, the collectivistic groups reported significantly greater social worth, belief changes and sharing of emotion than the individualistic group. Depression did not influence these cultural effects. Instead we found that those with depression, when compared to controls, considered emotions as subjective phenomena, that were qualifying for relationships with others, and associated with greater agency appraisals. The applicability of the biocultural framework of emotion in depression was considered.

## Introduction

Emotions are both universal and culture-specific^1–3^. That is, emotions are biologically determined products of our evolutionary past. Culture, in turn, regulates these biological emotions thereby influencing emotional responses to situations and the meaning given to different emotions^1^. Matsumoto and Hwang developed a biocultural framework of emotion that integrates the universality (biological-innateness) with cultural-specificity (cultural construction) of emotion^1^. Their framework considers priming reactions, subjective experience, and emotional meanings, and the relative influences of biology and culture on these domains of emotion^1^. Major depressive disorder (MDD) is a mood disorder characterized by disruptions in emotion. Researchers are increasingly attending to the influence of culture on emotion in depression^4,5^. However, to date, Matsumoto and Hwang’s framework has not been used to consider emotion in the context of depression.

Emotions have a considerable impact on people’s daily functioning and offer structure, continuity and purpose to our lives^6–8^. Thus, people often try to influence what they feel in order to match what they want to feel (i.e., ideal affect)^6–8^. However, there is significant variation in what people want to feel^6^. Although almost all people want to ‘feel good’, what is defined as ‘feeling good’ depends on the emotion states that are preferred, valued, and considered ideal; attitudes towards emotion; and norms regarding emotional experience^7,8^. Consequently, cultural factors play a particularly important role in determining how an individual defines ideal affect. Thus, ‘feeling good’ is a complex process influenced by the social-cultural context, timing of emotion, and individual motives and goals, which in turn impact mood-producing behaviors and cognitive efforts to regulate emotion^6–9^. Researchers have consequently highlighted the clinical need for research to investigate the interaction between individual and contextual factors in the experience of emotion^6–8^. This is especially pertinent to depression, given depression “changes the way we feel”^10^.

Matsumoto and Hwang provided a framework that integrates the individual and cultural contextual factors in emotion^1^. Specifically, in this framework, emotions are universally elicited in response to particular circumstances that are accompanied by universal and specific patterns of subjective feelings and physiological changes^1^. A core emotion system produces biologically innate emotions^11,12^. Matsumoto and Hwang’s model stresses, however, that emotions are not just biological. Rather, they are socially constructed and therefore need to be also understood on a social level of analysis^1^. Culture, a set of attitudes, norms, beliefs, behaviors and symbols shared by a group and transmitted across generations^13^, guide individual cognition, emotion and behavior allowing complex functioning in human social life^1^. Culture calibrates the core emotion system which regulates biological aspects of emotion ensuring emotions and associated reactions align with cultural norms. Cultural norms guide the regulation of emotion to ensure individual behavior adheres to culturally prescribed scripts, thereby, increasing social coordination^1^. In sum, culture functions to enable individuals to learn what to become emotional about, what kinds of reactions to have following an elicited emotion, how to regulate and express emotions, and the creation of values, cognitions and beliefs about emotion^1^.

Matsumoto and Hwang classify emotions into three domains; priming reactions, subjective experience and emotion meaning. Priming reactions are immediate emotional reactions to stimuli. They are highly dependent on a biologically innate processing system that activates some emotional states and has evolved over time to respond to human problems^1^. The biocultural framework assumes that when examining priming reactions of emotions, there will be greater contribution of biology and relatively less influence of culture, as these reactions are relatively immediate^1^.

Subjective experience is situated in an intermediary position concerning the influence of cultural and biological factors and requires some higher-order cognition that is beyond that required for priming reactions^1^. Whilst self-reports of biological emotions, especially if acquired in real-time, are considered universal, self-reports of cultural emotions are likely to vary depending on the cultural background of an individual. For instance, the construction and experience of emotions that are related to self-understanding are profoundly influenced by culture^14^. Western, individualistic cultures promote an independent self-construal in which uniqueness, personal control, and the pursuit of personal goals are paramount^15^. In collectivistic cultures, the emphasis is on interdependence, whereby, the self is characterized by a fundamental connectedness with others and a motivation to fulfil social obligations^15^. As almost all emotions are meaningful and often have relational themes^16^, emotions that align with these cultural models of self tend to be more prevalent, whilst emotions that are inconsistent tend to be experienced less frequently^3^. Therefore, cultural contexts supply meanings that in turn encourage certain themes resulting in systematic cultural variation in emotional experience^16^. For example, the interdependent self is associated with themes that promote relational and social harmony and seek to restore harmony when it has been temporarily disrupted. Emotions that engage these themes are considered interpersonally engaging. Engaging emotions are thus associated with, and affirm, the interdependent self. In contrast, other themes, such as personal achievement, unfair infringement of personal goals, and separateness from others, are emphasized by the independent self and are considered interpersonally disengaging^16^. In support of these assertions, individuals in collectivistic cultures report experiencing interpersonally engaging emotions more than interpersonally disengaging emotions. In contrast, those from individualistic cultures tend to experience more interpersonally disengaging emotions than engaging emotions^16–19^.

Emotion meaning refers to concepts, preferences and beliefs about emotions^1^. These processes require a high degree of language and higher order thinking^1^. In terms of creating meanings about emotion, there is the requirement of attitudes, cognitions, concepts, attitudes, beliefs and values (all of which are culturally dependent), resulting in this domain of emotion being highly influenced by culture when compared to the other domains (i.e., priming reactions and subjective experience)^1^. Therefore, Matsumoto and Hwang propose that emotion meanings have considerable cross-culturally variability^1^. In support of this premise, Mesquita found that members of collectivistic cultures (Turkish and Surinamese) perceived their emotions as relational phenomena and based their ratings of emotion in assessments of social worth, whilst participants in an individualistic culture (Dutch) focused more on the inner world of the individual^20^.

Those with MDD have difficulties in emotion. This includes altered emotion perception (including faces and body movement)^21^, emotion regulation^10^ and appraisals of emotion^22^. Indeed, models of depression highlight that depression is strongly associated with high negative emotion and low positive emotion and that an interaction of cognitive and emotional mechanisms underpin the development and maintenance of this disorder^10,23–27^. Understanding emotion in those with depression is therefore of utmost importance. Despite increasing understanding regarding the role of emotion in depression, relatively little is known about how culture influences emotion in those with depression. De Vaus and colleagues recently highlighted that cultural differences in how people think about emotion, experience emotion, and interpret and respond to emotion are likely to contribute to the development and maintenance of depression^27^. Specifically, their model posits that those from different cultures have different systems of thought about emotion and these cultural values, beliefs and thinking styles impact on emotion functioning, which in turn have specific consequences for emotion and cognition. Thus, culturally ingrained systems shape the way in which individuals think about and cope with emotions, thereby, influencing the impact of emotion on mental health^5^. De Vaus and colleagues highlight that there is a great need for cross-cultural comparisons of how people think about and experience emotion, as such investigations will enable us to better understand affective disorders^5^. Moreover, cross-cultural comparisons are important as major depressive disorder is an important contributor to the global burden of disease^28^. As this emerging area continues to develop, it is essential that theoretical models are utilized to guide empirical work.

Thus, the aim of the current study was to examine whether Matsumoto and Hwang’s biocultural model of emotion^1^ could be utilized in furthering our understanding of culture and emotion in depression. Specifically, we aimed to investigate the influence of culture on the three emotion domains (priming reactions, subjective meaning and emotion meaning) in depression in members from three different cultural groups; Australian (individualistic), Malay (collectivistic) and Iranian (collectivistic). Cross-cultural comparison studies tend to focus on Chinese or Japanese cultural groups (as an example of an Asian collectivistic culture) versus American (as an example of a Western individualistic culture) individuals. However, within individualistic and collectivistic societies there are variable patterns of societal change^29,30^. Hence, it is essential that research also considers examples of other individualistic and collectivistic cultures^30^. While Australian and American societies emphasize individualistic values, researchers have identified cultural differences in agency needs between these two cultural groups^31,32^. Most Asian and Middle Eastern cultures are collectivist^31–33^. However, the social emphasis of collectivism is influenced by other cultural factors which offer different meanings and consequences for the socially-defined self^34^. Unlike hierarchical Confucian societies of China and Japan, Malays derive their emphasis on collectivism from the traditional cultures of Islam and the indigenous Malay^34^. Iranian culture places value on tradition, religious Islamic beliefs, and conformity, but also promotes achievement and self-direction^33^. Thus, there is a need to investigate emotion in different individualist and collectivist cultures.

To investigate the influence of culture and depression on priming reactions we used a biological motion processing task. Humans have an excellent ability to obtain accurate perceptual information based on the shape of a human body moving in dim light in the distance^35^. In biological motion tasks participants are typically presented with stimuli that consist of moving point-light displays (PLDs) of actors. Participants tend to automatically perceive these events as social interactions and attribute particular emotions to these shapes^35^. Whilst these tasks assess an individual’s ability to recognize emotional states depicted by the PLDs, these tasks minimize structural cues and allow the separation of information revealed by motion from that derived from other sources^35^. Therefore, the use of biological motion has potential for the cross-cultural study of perceptual reactions to emotional stimuli as the task requires little verbal interaction and the context is culturally-neutral. Indeed, cross-cultural studies using biological motion have concluded that perception of biological motion is universal^36^. Research has demonstrated that those with MDD, when compared to healthy controls, display distorted emotion perception when rating emotions expressed via body movements depicted as PLDs^21,37^. Therefore, we hypothesized that those with MDD, regardless of cultural group, would have deficits in emotion perception when compared to controls (Hypothesis 1).

To examine subjective experience we employed a task previously used in the cross-cultural research and cited by Matsumoto and Hwang^1^ as an index of subjective emotional experience. Specifically, participants were required to report how intensely they had experienced interpersonally engaging and interpersonally disengaging emotions in recent situations^16^. It was predicted that participants in collectivistic cultures, when compared to those from individualistic cultures, would report experiencing significantly greater interpersonally engaging emotions (Hypothesis 2a) and fewer disengaging emotions (Hypothesis 2b)^16,17^. Moreover, well-being has been found to be more closely related to experiencing positive engaging emotions than with that of disengaging emotions for those from collectivistic cultures, whilst the reverse pattern has been found for those from individualistic cultures^16^. Recent models^38,39^ have acknowledged that violations of cultural norms and expectations play a role in psychopathology. Thus, we predicted that depression would interact with these cultural effects, whereby, those with depression in collectivistic cultures would report significantly less emphasis on interpersonally engaging emotions (given the cultural expectation of engaging emotion) than healthy controls (Hypothesis 3a), whilst those with depression in individualistic cultures would report less emphasis on interpersonally disengaging emotions (given the cultural expectation of disengaging emotion) than healthy controls (Hypothesis 3b).

Finally, we adopted Mesquita’s emotion meaning questionnaire^20^ to investigate different components of emotion. Members of collectivistic cultures tend to perceive their emotions as relational phenomena and ground their ratings in assessments of social worth, whilst members of individualistic cultures perceive emotions as less reflective of the social environment (*social worth*)^20^. Members of individualistic cultures tend to emphasize appraisals of agency when compared to those from collectivistic cultures (*appraisals*)^40^. Emotions in collectivistic cultures are generally about circumstances of shared concern and there tends to be agreed, unanimous validation of what these circumstances mean. Therefore, in collectivistic cultures the meaning of emotional situations is perceived as obvious, when compared to individualistic cultures, where emotions are perceived as subjective phenomena (*perceived source of appraisal*)^20^. Additionally, those in collectivistic cultures, when compared to individualistic cultures, perceive more permeable self-other boundaries resulting in greater *social sharing* of the emotion. Finally, collectivistic cultures tend to consider emotions as pieces of information that contribute to beliefs about the world, whilst individualistic cultures consider emotions as less pertinent to beliefs. Thus, emotions in collectivistic cultures tend to result in belief changes more often than in individualistic cultures (*belief changes*)^20^. We, therefore, predicted that those from collectivistic cultures, when compared to those from individualistic cultures, would report greater social worth (Hypothesis 4a), social sharing (Hypothesis 4b), perceived source of appraisal (Hypothesis 4c) and belief changes (Hypothesis 4d) and fewer agency appraisals (Hypothesis 4e). Additionally, as noted above, given recent models^38,39^ acknowledge that violations of cultural norms and expectations play a role in psychopathology, we predicted that depression would interact with these cultural effects; in each cultural group violations of these cultural norms would be evident in the depressed groups to a greater extent than in controls (Hypothesis 5).

## Results

### Data analysis plan

Statistical analyses were conducted using the Statistical Package for Social Sciences (SPSS) version 25. In order to examine Hypothesis 1, a 3 (group; Malay, Australia, Iran) x 2 (depression; MDD, control) analysis of variance (ANOVA) was conducted with proportion of correct responses on the biological motion task as the dependent variable. To examine Hypotheses 2 and 3, mean intensity ratings of the emotions that varied in social orientation (engaging, disengaging) were computed for each participant and submitted to an ANOVA with two between-subjects variables (culture and depression) and one within-subjects variables (social orientation). To examine Hypotheses 4 and 5, a series of 3 (group; Malay, Australian, Iranian) x 2 (depression; MDD, control) ANOVAs, with emotion meaning variables as the dependent variables, were conducted. Due to the sample size, we did not include valence or situation-type as variables in these analyses (see Supplemental Material for an overview of these post-hoc analyses). Partial eta-squared and Cohen’s d were used to report effect sizes for ANOVAs and *t* tests respectively, with sizes interpreted according to the framework provided by Cohen^41^.

### Group characteristics

Group characteristics are displayed in Table 1. The groups did not differ significantly in terms of gender, χ^2^ (*df* = 5, *N* = 146) = 6.07, *ns*, or age, *F*(5, 139) = 2.10, *ns*, *η*_*p*_^2^ = 0.07. A 3 (culture: Malay, Australian, Iranian) x 2 (depression: MDD, control) ANOVA with depression scores as the dependent variable revealed that, as expected, the depression main effect was significant, *F*(1, 140) = 207.07, *p* < 0.001, *η*_*p*_^2^ = 0.60. The culture main effect and interaction were both non-significant. To confirm that there were the expected cultural differences in our sample in independent/interdependent self-orientation, each participant received a self-construal ratio (ratio of independence divided by interdependence) and we compared groups on this ratio. This analysis revealed that although the Malay and Iranian groups did not differ, participants from the collectivistic cultures had a significantly lower proportion of independence relative to interdependence (*M* = 0.97, *SD* = 0.15) than did participants in the individualistic group (*M* = 1.05, *SD* = 0.25), *t*(144) = 2.58, *p* = 0.01, *d* = 0.39.

**Table 1:** Group Characteristics and Means (and Standard Deviations) for the Emotion Meaning Task.

	Malay		Iranian		Australian	
	MDD (*n* = 37)	Control (*n* = 37)	MDD (*n* = 15)	Control (*n* = 15)	MDD (*n* = 19)	Control (*n* = 23)
Age	31.65 (9.82)	28.95 (6.20)	35.53 (8.11)	32.20 (7.35)	27.06 (10.29)	35.52 (15.52)
Gender F:M	25:12	31:6	12:3	12:3	12:7	14:9
BDI-II	27.76 (11.50)	7.97 (5.32)	32.60 (10.36)	9.00 (7.30)	27.53 (10.00)	5.00 (4.91)
Emotion Recognition Task^a^	1.23 (0.19)	1.24 (0.17)	1.20 (0.21)	1.27 (0.23)	1.19 (0.14)	1.32 (0.14)
**Emotion Meaning**						
Social Worth	19.11 (8.35)	19.49 (6.69)	15.59 (4.72)	19.40 (7.62)	13.94 (8.77)	12.91 (7.86)
Appraisals	34.89 (4.78)	34.53 (5.17)	32.78 (5.03)	28.77 (5.72)	32.11 (5.80)	30.60 (5.26)
Source	35.92 (10.00)	38.35 (7.42)	31.00 (9.65)	38.93 (11.34)	42.56 (7.13)	45.35 (6.47)
Shared	11.18 (4.27)	14.16 (3.21)	11.29 (4.19)	15.27 (1.33)	5.63 (3.44)	4.30 (3.48)
Beliefs	56.54 (10.37)	46.30 (11.52)	41.69 (11.07)	42.67 (11.68)	52.16 (12.31)	35.87 (11.34)

Note: BDI-II = Beck Depression Inventory-II; MDD = major depressive disorder. Source = source of appraisals; shared = shared emotion; beliefs = belief changes. ^a^Proportion of Correct Responses on Emotion Recognition Task.

### Hypothesis 1: Emotion Recognition (PLD)

Table 1 presents the mean proportion correct responses for each group’s performance on the PLD task. As predicted the depression main effect was significant, *F*(1, 140) = 4.72, *p* = 0.03, *η*_*p*_^2^ = 0.03; the MDD group had a lower proportion of correct responses than the control group (Hypothesis 1). The culture main effect, *F*(2, 140) = 0.15, *p* = 0.86, *η*_*p*_^2^ < 0.01 and interaction, *F*(2, 140) = 1.52, *p* = 0.22, *η*_*p*_^2^ = 0.02, were non-significant.

### Hypotheses 2 and 3: Subjective Emotional Experience

**Figure 1:**
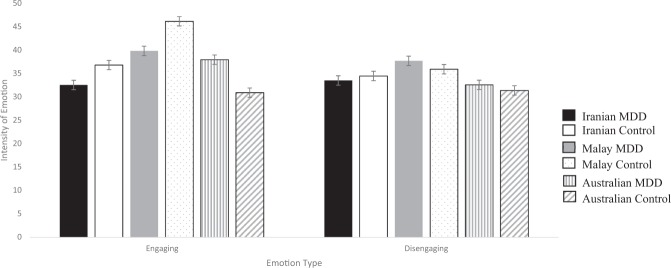
Mean (±SE) of Subjective Experience for Engaging and Disengaging Emotion.

Figure 1 presents the mean intensity ratings of interpersonally engaging and disengaging emotion for each of the groups. As hypothesized, there was a significant interaction involving culture, depression and social orientation of the emotion, *F*(2, 140) = 5.72, *p* < 0.01, *η*_*p*_^2^ = 0.08. Follow-up analyses found that for interpersonally engaging emotion the culture x depression interaction was significant, *F*(2, 140) = 3.34, *p* = 0.04, *η*_*p*_^2^ = 0.05. The control groups differed significantly, *F*(2, 72) = 10.74, *p* < 0.001, *η*_*p*_^2^ = 0.23; the Malay control group reported significantly greater interpersonally engaging emotion than the Iranian control group, *t*(50) = 2.23, *p* = 0.03, *d* = 0.68, 95%CI[0.06–1.28], and Australian control group, *t*(58) = 4.23, *p* < 0.001, *d* = 1.12, 95%CI[0.55–1.67]. The Iranian group scored at the intermediate level and tended to score higher than the Australian group, *t*(36) = 1.94, *p* = 0.06, *d* = 0.64, 95%CI[−0.03–1.30] (Hypothesis 2a). However, the three depressed groups did not differ significantly, *F*(2, 68) = 1.27, *p* = 0.29, *η*_*p*_^2^ = 0.04. While interpersonally engaging emotion did not differentiate between those with and without MDD for the Iranian group or Malay group, when we considered the collectivistic group as a whole, the collectivistic MDD group reported fewer engaging emotions than the collectivistic control group, *t*(102) = 1.97, *p* = 0.05, *d* = 0.39, 95%CI[0.14–1.46] (Hypothesis 3a). The Australian MDD group reported significantly greater engaging emotion than the Australian control group, *t*(40) = 2.04, *p* < 0.05, *d* = 0.63, 95%CI[0.001-0.1.24]. For interpersonally disengaging emotion, the cultural group main effect, *F*(2, 140) = 2.03, *p* = 0.14, *η*_*p*_^2^ = 0.03, depression main effect, *F*(1, 140) = 0.09, *p* = 0.76, *η*_*p*_^2^ = 0.001, and culture x depression interaction, *F*(2, 140) = 0.13, *p* = 0.88, *η*_*p*_^2^ < 0.01, were all non-significant (Hypothesis 2b and Hypothesis 3b).

### Hypotheses 4 and 5: Emotion Meaning

Table 1 presents the means for the emotion meaning variables. In terms of time since the event, it was found that the main effects and interactions were all non-significant.

#### Concerns: social worth

In support of our hypothesis, the culture main effect was significant, *F*(2, 140) = 7.90, *p* = 0.001, *η*_*p*_^2^ = 0.10. While the Iranian and Malay groups did not differ significantly, *t*(104) = 1.26, *ns*, *d* = 0.27, 95%CI[−0.68–0.15], both the Iranian group, *t*(70) = 2.26, *p* = 0.03, *d* = 0.54, 95%CI[0.06–1.00], and Malay group, *t*(112) = 3.89, *p* < 0.001, *d* = 0.76, 95%CI[0.36–1.15], reported significantly greater social worth than the Australian group (Hypothesis 4a). However, contrary to Hypothesis 5, depression did not influence this cultural effect, *F*(2, 140) = 0.97, *p* = 0.38, *η*_*p*_^2^ = 0.01, and the depression main effect was also non-significant, *F*(1, 140) = 0.63, *p* = 0.43, *η*_*p*_^2^ < 0.01.

#### Shared emotion

As predicted, the culture x depression interaction was significant, *F*(2, 142) = 6.41, *p* < 0.01, *η*_*p*_^2^ = 0.08. The Iranian groups did not differ significantly from the Malay groups; control, *t*(50) = 1.28, *p* = 0.21, *d* = 0.39, 95%CI[−0.22–0.99], depressed, *t*(52) = 0.08, *p* = 0.93, *d* = 0.02, 95%CI[−0.55–0.60]. The Australian groups scored significantly lower than the collectivistic groups; Iranian control vs Australian control, *t*(36) = 11.60, *p* < 0.001, *d* = 3.86, 95%CI[2.71–4.85]; Iranian depressed vs Australian depressed, *t*(34) = 4.45, *p* < 0.001, *d* = 1.49, 95%CI[0.72–2.19]; Malay control vs Australian control, *t*(58) = 11.20, *p* < 0.001, *d* = 2.97, 95%CI[2.20–3.68]; Malay depressed vs Australian depressed, *t*(54) = 4.91, *p* < 0.001, *d* = 1.39, 95%CI[0.76–1.98] (Hypothesis 4b). In support of Hypothesis 5, Malays and Iranians with MDD shared the event significantly less than controls; Malay, *t*(72) = 3.39, *p* = 0.001, *d* = 0.79, 95%CI[0.31–1.25]; Iranian, *t*(30) = 3.51, *p* = 0.02, *d* = 1.25, 95%CI[0.46–1.97]. For the Australian group, the sharing of the emotion did not differentiate between those with and without MDD, *t*(40) = 1.24, *p* = 0.22, *d* = 0.38, 95%CI[−0.99–0.24].

#### Source of appraisal

The culture main effect was significant, *F*(2, 140) = 11.50, *p* < 0.001, *η*_*p*_^2^ = 0.14; while the Iranian and Malay groups did not differ significantly, *t*(103) = 1.20, *p* = 0.23, *d* = 0.25, 95%CI[−0.16–0.67], the Australian group scored significantly higher than the Malay group, *t*(112) = 4.38, *p* < 0.001, *d* = 0.86, 95%CI[0.45–1.24], and Iranian group, *t*(71) = 4.47, *p* < 0.001, *d* = 1.06, 95%CI[0.56–1.53] (Hypothesis 4c). Contrary to Hypothesis 5, depression did not influence this culture main effect, *F*(2, 140) = 1.20, *p* = 0.31, *η*_*p*_^2^ = 0.02. Rather pan-culturally source of appraisal was significantly greater for the control group than MDD group, *F*(1, 140) = 8.22, *p* < 0.01, *η*_*p*_^2^ = 0.06 (i.e., depression main effect).

### Belief changes

The culture x depression interaction was significant, *F*(2, 141) = 5.26, *p* < 0.01, *η*_*p*_^2^ = 0.07. The Iranian and Malay control groups did not differ significantly, *t*(50) = 1.03, *p* = 0.31, *d* = 0.31, 95%CI[−0.91–0.29]. The Malay control group scored significantly higher than the Australian control group, *t*(58) = 3.43, *p* = 0.001, *d* = 0.91, 95%CI[0.35–1.44], but the Australian and Iranian control groups did not differ significantly, *t*(36) = 1.79, *p* = 0.08, *d* = 0.59, 95%CI[−0.08–1.24] (although a medium effect size was observed) (Hypothesis 4d). The Iranian MDD group scored significantly lower than the Malay MDD group, *t*(51) = 4.69, *p* < 0.001, *d* = 1.40, 95%CI[0.74–2.03], and Australian MDD group, *t*(33) = 2.62, *p* = 0.01, *d* = 1.35, 95%CI[0.59–2.05]. The Australian and Malay MDD groups did not differ significantly, *t*(54) = 1.41, *p* = 0.17, *d* = 0.40, 95%CI[−0.17–0.95]. In support of Hypothesis 5, Australian MDD participants reported significantly greater belief than Australian controls, *t*(40) = 4.46, *p* < 0.001, *d* = 1.38, 95%CI[0.68–2.03]. Contrary to Hypothesis 5, however, Malay depressed participants also reported significantly greater belief changes than Malay controls, *t*(72) = 4.02, *p* < 0.001, *d* = 0.93, 95%CI[0.45–1.40], and belief changes did not differentiate between Iranians with and without MDD, *t*(29) = 0.24, *p* = 0.81, *d* = 0.09, 95%CI[−0.79–0.62].

#### Appraisals

The culture main effect was significant, *F*(2, 140) = 8.81, *p* < 0.001, *η*_*p*_^2^ = 0.11. There was no support for Hypothesis 4e, the Australian and Iranian groups did not differ significantly, *t*(70) = 0.32, *p* = 0.75, *d* = 0.09, 95%CI[−0.56–0.37], and the Malay group scored significantly higher than the Iranian group, *t*(103) = 3.50, *p* = 0.001, *d* = 0.75, 95%CI[0.31–1.18], and Australian group, *t*(113) = 3.44, *p* = 0.001, *d* = 0.67, 95%CI[0.27–1.06]. There was no evidence to suggest that depression influenced this cultural main effect, *F*(2, 140) = 1.35, *p* = 0.26, *η*_*p*_^2^ = 0.02 (Hypothesis 5). Rather, the depression main effect was significant, *F*(2, 140) = 4.51, *p* = 0.04, *η*_*p*_^2^ = 0.03; those with MDD had significantly greater agency (i.e., control and responsibility) appraisals than the control group.

## Discussion

This study aimed to assess whether Matsumoto and Hwang’s biocultural model of emotion^1^ could be utilized in furthering our understanding of culture and emotion in depression. First, our findings offer support for the utility of this model in guiding culture and emotion research in the context of depression. Second, as predicted, those with depression, regardless of cultural background, had a lower proportion of correct responses on the emotion recognition of biological motion than healthy controls. Third, regarding subjective experience, as hypothesized, the Malay control group reported significantly greater socially-engaging emotion than the Australian control group, with the Iranian control group scoring at the intermediate level. Interestingly, the three depressed groups did not differ significantly. The collectivistic depressed group tended to report fewer engaging emotions than the collectivistic control group, whilst the Australian depressed group reported significantly greater engaging emotions than the Australian control group. Contrary to our hypotheses, for interpersonally disengaging emotion the culture and depression interaction was non-significant. Fourth, in terms of emotion meaning, as hypothesized, those in collectivistic cultures reported greater social worth, social sharing, and belief changes than those from individualistic cultures. However, there was little support for Hypothesis 5; only cultural violations of shared emotion differentiated between those with and without depression. Instead, contrary to Hypothesis 5, our findings tended to indicate that both depression and culture independently influenced emotion meaning.

Matsumoto and Hwang’s biocultural model of emotion^1^ was developed to guide researchers in the study of culture and emotion. The current study indicates that this model has potential utility in structuring future cross-cultural depression research. Matsumoto and Hwang claim that there is a universal biologically innate processing system that activates some emotional states; priming reactions. Such a system has evolved to deal with human-specific problems associated with survival, such as the effortless and universal perceptual ability of humans to attribute particular emotions based on the outline of a human body^35,36^. Those with depression have deficits in areas of social cognition, which extend to the perception of emotional body movements^21^. We found that those with depression, regardless of cultural background, had a lower proportion of correct responses on the emotion recognition biological motion paradigm than healthy control groups. This offers preliminary support for depression influencing the perceptual ability to determine social interactions and attribute particular emotions. Therefore, future research in the area is required especially as the study supports the use of PLDs in investigating emotion in psychiatric disorders as an exciting emerging area of research^42^.

Culture calibration of this core emotion system results in aspects of the subjective experience of emotion being culturally influenced^1^. Subjective experience is influenced by both biological and cultural factors, with self-reports of emotion recalled from memory being particularly culturally variable^1^. In support of this stance, and previous research^16–19^, we found that the collectivistic control group reported greater engaging emotions than the individualistic control group. However, depression influenced these cultural effects. Namely, for the depressed groups these cultural differences were not evident. Interpersonally engaging emotions are emphasized in collectivistic cultures, but are less valued in individualistic cultures. Violations of cultural norms and expectations play a role in psychopathology^38,39^. Thus, in this instance, a downplaying of engaging emotions in collectivistic cultures and an emphasis of engaging emotions in individualistic cultures, which contradicts cultural norms and expectations, were associated with depression. It is important to highlight here, however, that whilst we adopted a paradigm suggested by Matsumoto and Hwang^1^ as a paradigm that explores subjective emotion, the task assessed memory of the subjective experience. There is a significant difference between the experiencing of emotion and the memory of subjective experience of emotion, especially as culture has an influence on memory^43^. Further, time frame matters, as greater cultural differences have been observed for longer time frames than shorter time frames^44^. Thus, these finding need to be interpreted with caution and future research should examine actual subjective experience (i.e., real time versus over time^44^) through more ecologically valid methods, such as ambulatory assessment. We found no evidence of the expected cultural differences in disengaging emotion or evidence to indicate that culture and depression interacted to influence disengaging emotion. Previous studies have found mixed findings in terms of culture and social orientation of emotion^17^. Kitayama and colleagues suggest that mixed findings may reflect cultural differences in the manner in which engaged and disengaged emotion are constructed^17^. The lack of cultural differences may also reflect the different cultural groups employed in the current study, where previous studies have predominately used Japanese and European Americans^45^.

Culture is proposed to have a considerable influence on emotion meaning^1^. Our findings support this. Those in collectivistic cultures, when compared to individualistic cultures, had greater emphasis on relational concerns, more permeable self-other boundaries resulting in greater social sharing, and tended to treat emotions as pieces of information that fed into beliefs about the world^20^. There was no support for our cultural hypothesis for appraisals or perceived source of appraisal. In Mesquita’s study a considerable number of participants from both the collectivistic and individualistic groups indicated they did not assume the presence of an obvious, general norm of interpretation (i.e., perceived source of appraisal)^20^. Additionally, Mesquita did not find the expected cultural effects for all situation types^20^. Thus, differences in findings may relate to measurement differences or concerns regarding the conceptualization of this variable.

The only instances in which depression and culture interacted to influence emotion meaning was shared emotion and belief changes. As social sharing is culturally valued in collectivist groups to a greater extent than in individualist groups, a violation of this cultural norm was associated with depression for the collectivistic groups. However, for belief change, there was no clear support for our hypothesis. Whilst, as predicted, the Australian depressed participants reported significantly greater belief changes (i.e., an emotion meaning that violated individualistic cultural expectations) than controls, the Malay depressed participants also reported significantly greater belief changes than controls and beliefs changes did not differentiate between Iranians with and without depression. Instead, contrary to Hypothesis 5, we predominantly found that depression did not interact with culture to influence emotion meaning. Thus, overall there was little support for Hypothesis 5, with the depression x culture interactions for social worth, source of appraisal and appraisals being non-significant, with small effect sizes observed. Rather, those with depression, when compared to controls, tended to have greater relational concerns (social worth) and agency (i.e., control, responsibility) appraisals and perceived emotion as subjective phenomena.

Overall our findings suggest that in line with Matsumoto and Hwang’s model^1^ depression may affect emotion at the priming level in universally similar ways. At the intermediate, subjective experience level, culture and depression may interact to influence the recall of emotion, especially self-reports of emotion related to self-understanding. Thus, at this level there may be aspects in which the biological and cultural influences of emotion in depression interact. In terms of emotion meaning, we did not find strong evidence to suggest that culture interacted with depression in influencing emotion meaning. Instead we tended to find culture main effects and depression main effects, which suggest both culture and depression may independently influence emotion meaning. Thus, it possible that at this level culture has such strong influences on this emotion domain that these cultural main effects persist regardless of depression status. However, further research is required to investigate these hypotheses.

It is important to also situate our findings within the broader emotion and culture literature. First, as De Vaus and colleagues recently posited, culture influences how people think about emotion, experience emotion and interpret emotion, which in turn contributes to depression^5^. Our findings offer initial support for these claims and demonstrate that cross-cultural frameworks of emotion can be used to guide empirical work in the clinical area of depression. Whilst our findings are preliminary, they highlight a need for further research examining emotion and culture in depression. In terms of the emotion literature, it is consistently emphasized that there is a complex interaction between cultural, situational, timing and individual factors in influencing emotion^6–8^. Thus, the factors influencing the three levels of Matsumoto and Hwang’s biocultural model are complex and extend beyond culture and depression. For instance, Scollon and colleagues highlight that cultural norms shape emotional experiences to varying degrees depending on the valence of emotion, the specific emotion being examined, and time frame of the emotional experience^44^. Tsai highlights the need to consider the interaction of cultural and temperamental factors in shaping emotion and behavioral consequences^7,8^. Furthermore, emotion regulation strategies need to be considered within the situation, timeframe, specific emotion being examined and motivation of the individual^6,9^. In sum, while Matsumoto and Hwang’s biocultural model has utility in the context of culture and depression, there also needs to be incorporation of the complexity of the experience, timing, valence and temperamental factors. As Scollon and colleagues claim, while it is well-established that there is considerable intercultural variability in self-reports of emotion, greater attention is needed in terms of understanding when and why and these differences occur, taking into account valence, motivation and regulation factors^44^.

The aim of this study was to initiate an investigation of the utility of Matsumoto and Hwang’s biocultural model^1^ in depression research. Thus, the tasks and constructs (i.e., self-construal) employed in the current study matched that referred to in Matsumoto and Hwang’s article. Individuals were from different nationalities that have been shown to be either individualistic (Australia) or collectivistic (Malay, Iran)^46^. These groups differed significantly in terms of proportion of independence relative to interdependence. We adopted this approach because previous culture and emotion research has also used this approach^16,17,20^, and this approach is routinely used in cross-cultural clinical research^47–49^. However, this approach it is not without limitations. Specifically, it assumes that these proxies for individualism-collectivism are consistent over time, situations, and life domains^50^. Additionally, the groups differ on a range of other cultural dimensions, which may have influenced findings. The next stage of research, therefore, is to include greater assessment of cultural norms, such as independence/interdependence^15^ or holistic/analytical orientations^51^ and to examine explicitly measured cultural variables at the individual level (e.g., using a portfolio of self-construal, acculturation, and other measures) and to correlate this assessment with individual outcomes of emotion. This would allow for greater clarification concerning the influence of culture on emotion in depression.

The research has several potential clinical implications. First training programs that target emotion priming reaction deficits in depression (e.g., cognitive bias modification training etc.)^52^ may have cross-cultural utility. Second, socially oriented emotion associated with mundane events may culturally differ. Thus, clinicians may need greater understanding of these cultural differences and how to assess and target culturally differing subjective emotions in the treatment for depression. For instance, those with individualistic values may need to downplay interpersonally engaging emotion, whilst there may need to be a greater emphasis on increasing interpersonally engaging emotion in those from collectivistic cultures. Finally, culture is considered to have considerable influence on the meaning given to emotion; a target of depression interventions. However, at this point, further research is required before firm conclusions can be drawn regarding how these important considerations can be used to inform practice.

There are several limitations worth noting. First, the cross-section design prevents causal inferences. Second, the unequal sample sizes may have influenced findings and our sample size was modest. Although the sample size met pre-study targets derived from power analyses of existing data for the Australian and Malay groups, the analyses employing the Iranian samples may have been underpowered. However, we included the Iranian data, as it is important to focus on collectivistic cultures other than those routinely included in the literature (i.e., Japan and China)^45^. Thus, these analyses and the interaction findings in particular need to be considered somewhat preliminary. Third, as noted above, the sample was based on nationality and thus, future research should include greater assessment of cultural norms and explicitly measured cultural variables at the individual level. The fourth issue concerns comorbidity. The study excluded participants with PTSD, an anxiety disorder and bipolar disorder. This offers some confidence that the current findings are not merely the function of co-morbidity variables^53^. However, many participants in the depressed group reported anxiety symptoms. Furthermore, given clinical depressed groups frequently present with co-morbid anxiety, future studies would benefit from the inclusion of measures of state and trait anxiety to improve clinical generalizability particularly as emotional disruptions are transdiagnostic (i.e., not disorder specific). Thus, at this stage we cannot be certain of the influence of comorbidity factors in accounting our findings, or interacting with depression, and this needs to be considered when interpreting our findings. Fifth, the cultural groups in the current study differed to those routinely used in the culture and emotion research (i.e., Japanese and European American). This is a strength of our study as research in this area needs to expand beyond these two cultural groups^45^. However, the theoretical foundations on which the study was developed may need to be considered in light of this difference in cultural samples. Sixth, the measures included in this study have been developed for cross-cultural emotion research and internal reliability was found to be good. However, a limitation of cross-cultural research is that the variables may not be working in a similar way across cultural groups. We conducted exploratory analyses of the data using SPSS and AMOS (i.e., in the collectivistic group and individualistic groups we explored the behavior of the variables, quality of the data, and exploratory factor analyses)^54^, which indicated that our emotion variables were working in a similar way across cultural groups. However, future studies with larger sample sizes are needed to ascertain measurement invariance. Seventh, as noted above, the study assessed memory of subjective experience rather than actual subjective experience of emotion. Future research should employ ecologically valid measures of subjective emotion in depression.

Despite these limitations, this was the first study to apply Matsumoto and Hwang’s model of culture and emotion^1^ to depression. The findings suggested that this model has utility in guiding future research.

## Method

### Participants

Participants were Iranian (*n* = 32), European Australian (*n* = 42), and Malay (n = 74) individuals with current depression and never-depressed, healthy controls living in their country of origin. Australia is considered an individualistic culture, whereas Iran and Malaysia are considered collectivistic societies (Iran is rated less collectivistic than Malaysia)^46^. Participants were recruited from the general community by advertisements in local newspapers and social media, posters in public places, and contacts with organizations that provide treatment for depressed patients. Participants were included if they met the eligibility criteria, which included appropriate cultural background (i.e., both grandparents of either European Australian descent, Malay descent, or Iranian descent); aged between 18 and 60 years; and no history or current diagnosis of substance dependence, bipolar disorder, an anxiety disorder, posttraumatic stress disorder, psychosis, or organic brain injury. We excluded those with an anxiety disorder given theoretical models highlight that depression and anxiety have overlapping and distinctive affective features^27,55^. Given this was the first study to investigate Matsumoto and Hwang’s biocultural model^1^ in clinical affective science research, we decided to focus on depression. The control group were also required to have no history or current diagnosis of depression or any other mental health disorder. These eligibility criteria were assessed over the telephone using the Structured Clinical Interview for the Diagnostic and Statistical Manual of Mental Disorders–Fifth Edition, Research Version Screening Module (SCID-5-RV)^56^. Eligible participants attended a testing session in which the SCID-5 was administered by LJ (Australia), VM (Iran) and SM (Malaysia). All interviews were audio-recorded and 25% of the interviews were coded by clinical psychologists independent of the research team, and thus, blind to participant group and hypotheses. There was complete agreement between raters. Participants were allocated to the currently-depressed or never-depressed control groups based on the SCID-5.

Given the novelty of the study, it was difficult to calculate a-priori sample size estimates. In the cross-cultural research, medium to large effect sizes have tended to be observed regarding emotional experience and appraisals^16,20^. Sample size considerations for this study were also based on a review of recent cross-cultural clinical comparison studies^47,48,57^. Based on these reviews, we adopted a medium effect size, with an alpha of 0.05, and 80% power, which indicated that at least 19 participants per group was required, which is a similar sample size to that used in previous cross-cultural clinical research. Furthermore, due to the modest sample size, our analyses were only hypothesis-focused (i.e., we did not include the interactive effects of valence or situation in our analyses), rather Supplemental Material provides an overview of these post-hoc analyses.

### Measures and Materials

#### Biological motion task

We adopted the biological motion paradigm and stimuli used in previous research^58^. Stimuli consisted of moving PLDs (white dots against a black background) of female and male actors performing either: knocking, walking, jumping on the spot, or kicking a ball. Participants watched a series of short movies (144 movie trials; duration of 3 s). Each trial included a ‘prime’ PLD movie, which was followed by a ‘target’ PLD movie. While the two movies (prime and target) were the same in terms of the gender of the figure and action performed, the viewing perspective always differed (e.g., if the prime was viewed from the side view, the target was presented from the 45° view). Viewing perspective changed in order to increase task-difficulty to ensure participants were identifying and interpreting movement kinematics and not merely comparing lower-order visual properties^58^. In the prime-movie a point-light figure in the ‘neutral emotional state’ was always shown, whilst in the target movies the point-light figure could be either positive (happy), negative (sad, angry) or neutral. Participants were provided with verbal and visual standardized instructions requesting them to indicate as quickly as possible whether the PLD figure in the ‘target’ movie performed the action in a different ‘emotional state’ than the PLD figure in the ‘prime’ movie. These response options (i.e., happier, sadder, angrier, or not different) were specified on response buttons on the computer keyboard. Accuracy rates (% correct answers) for positive, negative and neutral stimuli were assessed for all participants.

#### Subjective experience questionnaire

We used a previously and commonly used approach to investigate interpersonally engaging and interpersonally disengaging emotion^16,18^. Participants were asked to remember “the most recent event of 10 different situations and asked to rate the emotions you experienced in each of these situations”. The situations were positive (positive interaction with friend, good interaction with family members, heard a positive comment about appearance, watched TV or listened to music, engaged in a sporting activity) and negative (being caught in a traffic jam, overloaded with work, getting ill or injured, problem with family member, argument of problem with friends)^16^. They were asked to briefly think about the episode and then to report how strongly they had experienced each of 14 emotions. The ratings were made on a 6-point scale ranging from 0 (*not experienced it at all*) to 5 (*experienced it very strongly*). All engaging and disengaging emotion terms have been extensively used in previous research^16–18^; positive engaging (friendly feelings towards others, sympathy for another, feelings of closeness to others), positive disengaging (top of the world, proud, superior, self-esteem), negative engaging (guilt, ashamed, indebted, afraid of causing trouble on another) and negative disengaging (sulky feelings, frustrated, angry). The total for each emotion term was calculated for each participant and divided by the number of emotions contained in each emotion term. Internal consistency for these emotion domains ranged from α = 0.92-0.96.

#### Emotion meaning questionnaire

We adopted the Mesquita’s questionnaire^20^, an approach classified by Matsumoto and Hwang as an index of emotion meaning^1^. Participants were required to answer questions in relation to two stimulus events (i.e., significant positive event, significant negative event). Participants were asked to report an instance from the past that fits the two stimulus events and, subsequently, to answer questions pertaining to this event. The subscales of the questionnaire assessed social worth, appraisals, source of appraisals, social sharing of emotions, and belief changes. The questions included were drawn from Mesquita’s questionnaire^15^. *Social worth* was assessed by four questions (rated on a 9-point scale ranging from 1 = *not at all* to 9 = *extremely*) designed to test change in social worth: respect, prestige, family respect and in-group. *Appraisal* scales were rated on a 9-point scales (ranging from 1 = *not at all* and 9 = *extremely*) and included items that are considered to be agency-focused appraisals (i.e., focused on control and responsibility)^3,57^. Responses were tallied to give a total independent appraisal score. *Source of appraisal* was measured using three questions (rated on a 9-point scale ranging from 1 = *not at all* to 9 = *extremely*) assessing the obviousness of meaning and implications (i.e., asking whether another person would find the situation as pleasant or unpleasant as the respondent did, would think and feel in a similar way, and would react similarly to the way the respondent had). *Social sharing of emotions* questions related to the first time the emotional experience was shared with someone else. Participants responded to these nine items on dichotomous responses (1 = *no* and 2 = *yes*). The total number of ‘yes’ responses was tallied for each participant to provide an index of social sharing of emotion, with higher scores indicating greater sharing of emotion. *Belief changes* were assessed using a four-item scale, in which items were rated on 9-point scales (1 = *not at all* to 9 = *extremely*) measuring changes in self-confidence, behavior, self-respect, and motivation. Internal consistency for these subscales ranged from α = 0.70-0.86.

### Translation

Tasks and measures were administered in Malay, Farsi and English. The original English tasks and measures were forward-translated into Malay or Farsi by SM and VM (a Malay native speaker and a Farsi native speaker both fluent in English). The Malay and Farsi versions were then back-translated into English by a third independent translator who was blind to the original English instruments. Authors (FM and AM) then examined the translated versions in order to detect and resolve any ambiguities. The study paradigm was piloted on a convenience sample in each culture.

### Procedure

Ethical approval was obtained from Monash University (Australia), Kharazmi University (Iran) and Universiti Putra (Malaysia). We confirm that the research was conducted in accordance with established American Psychological Association ethical guidelines. Following informed consent, participants completed the SCID-5. Participants then completed demographics, the Beck Depression Inventory-II^59^, which was used to assess depression, and the Self Construal Scale^60^, as a measure of individualism and collectivism. Following this, participants completed the biological motion task on a computer, in which participants sat approximately 50 cm from the computer screen. Finally, participants completed the subjective experience and emotional meaning questionnaires. Participants were reimbursed $A30 for their time.

## Supplementary information

Supplemental Information: Overview of post-hoc analyses
