# Supplementary material for: Investigating Emotion in Malay, Australian and Iranian Individuals with and without Depression
Source: Sci Rep. 2019 Dec 4;9:18344. doi: 10.1038/s41598-019-54775-x (PMC6892853; doi:10.1038/s41598-019-54775-x)
Supplement: Supplementary file 1 — Supplemental Information: Overview of post-hoc analyses [file 41598_2019_54775_MOESM1_ESM.docx]

Investigating Emotion in Malay, Australian and Iranian Individuals with and without Depression

Laura Jobson

Monash Institute of Cognitive and Clinical Neurosciences, Monash University, Melbourne Australia

Vida Mirabolfathi

Kharazmi University and Institute for Cognitive Science Studies, Tehran, Iran

Shiva Moshirpanahi

Kharazmi University, Tehran, Iran

Hadi Parhoon

Kharazmi University, Tehran, Iran

Julia Gillard

University College London, UK

Firdaus Muhktar^*^

Universiti Putra, Kuala Lumpur, Malaysia

Ali Reza Moradi^*^

Kharazmi University and Institute for Cognitive Science Studies, Tehran, Iran

Sindhu Nair Mohan

Universiti Putra, Kuala Lumpur, Malaysia

^*^ Corresponding authors:

Firdaus Muhktar Psychiatry Department, Universiti Putra Malaysia, Malaysia.

Phone:+60389472543 Fax:+60389472621 Email:[drfircbt@gmail.com](mailto:drfircbt@gmail.com)

Ali Reza Moradi Kharazmi University & Institute for Cognitive Science Studies, Tehran, Iran. Email: [moradi@khu.ac.ir](mailto:moradi@khu.ac.ir).

All authors declare that they have no conflict of interest or competing financial or non-financial interests. LJ, SM, JG, FM and ARM designed the study. LJ was responsible for data collection in Australia. VM, SM, HP and ARM were responsible for data collection in Iran. SNM and FM were responsible for the data collection in Malaysia. All authors contributed to data analysis. LJ wrote the main manuscript text. All authors reviewed the manuscript.

**Supplemental Information: Overview of post-hoc analyses**

**Hypothesis 1: Emotion Recognition (PLD)**

Figure 1 presents the mean proportion correct responses for each group’s performance on the PLD task taking into account valence. The valence main effect was significant, *F*(2, 280)= 301.30, *p*< .001, *η_p_^2^*= .68; performance was significantly better for neutral images than negative, *t*(145)= 6.59, *p*< .001, *d*= 0.86, 95%CI[.62-1.10], and positive images, *t*(145)= 24.80, *p*< .001, *d*= 3.01, 95%CI[2.67-3.34]. Performance was also significantly worse for positive images than negative images, *t*(145)= 24.80, *p*< .001, *d*= 2.50, 95%CI[2.19-2.80]. The valence x depression interaction, *F*(2, 280)= .48, *p*= .62, *η_p_^2^*= .003, the valence x culture interaction, *F*(4, 280)= 1.42, *p*= .23, *η_p_^2^*= .02, and valence x culture x depression interaction, *F*(4, 280)= .62, *p*= .65, *η_p_^2^*= .01, were all non-significant.

**Hypotheses 2 and 3: Subjective Emotional Experience**

The significant interaction involving culture x depression x social orientation of the emotion, reported in the manuscript, was not influenced by situation type or valence. For interpersonally disengaging emotion, when considering valence and situation type, cultural differences were evident for positive disengaging emotion for the negative situation, whereby the Malay (*M*= 27.20, *SD*= 20.65) and Iranian (*M*= 33.70, *SD*= 20.75) groups did not differ significantly, *t*(102)= 1.45, *ns*, *d*= 0.31, 95%CI[-0.11-0.74], but contrary to that predicted, the Malay group, *t*(110)= 2.41, *p*= .02, *d*= 0.48, 95%CI[0.08-0.87], and Iranian group, *t*(66)= 3.43, *p*= .001, *d*= 0.84, 95%CI[0.33-1.33], scored significantly higher than the Australian group (*M*= 17.75, *SD*= 17.65). There was also a depression x valence interaction, *F*(1, 134)= 50.99, *p*< .001, *η_p_^2^*= 0.28. The control group reported significantly greater positive disengaging emotion (*M*= 76.03, *SD*= 33.07) than negative disengaging emotion (*M*= 45.56, *SD*= 22.68), *t*(74)= 7.11, *p*< .001, *d*= 1.07, 95%CI[0.73-1.41]. In contrast, the MDD group reported significantly greater negative disengaging emotion (*M* = 70.66, *SD* = 28.67) than positive disengaging emotion (*M* = 52.38, *SD* = 32.01), *t*(64) = 3.56, *p* = .001, *d* = 0.60, 95%CI[0.25-0.95]. The MDD group reported significantly greater negative disengaging, *t*(138)= 5.78, *p*< .001, *d*= 0.98, 95%CI[0.62-1.32], and significantly less positive disengaging emotion, *t*(138)= 4.28, *p*< .001, *d*= 0.73, 95%CI[0.38-1.06], than the control group.

**Hypotheses 4 and 5: Emotion Meaning**

Supplemental Table 1 presents the means for the emotion meaning variables taking into account situation type.

**Concerns: Social worth**

The situation type main effect was significant, *F*(1, 140)= 69.57, *p*< .001, *η_p_^2^*= 0.33; with social worth being significantly higher for the positive event than negative event. The situation type x depression interaction was significant, *F*(1, 140)= 5.22, *p*< .05, *η_p_^2^*= 0.04. Specifically, while the depressed and control groups did not differ significantly for the negative event, the controls scored significantly higher for the positive event than the depressed group, *t*(145) = 2.11, *p* = .04. The culture x situation type interaction and three-way interaction were non-significant.

**Shared emotion**

The situation type main effect, depression x situation type interaction, culture x situation type interaction and three-way interaction were non-significant.

**Source of appraisal**

The situation type main effect was significant, *F*(1, 140)= 6.01, *p*= .02, *η_p_^2^*= 0.04; with source of appraisal being significantly higher for the positive event than negative event. The situation type x depression; situation type x culture and three-way interaction were all non-significant.

**Belief changes**

The situation type main effect was significant, *F*(1, 141)= 27.07, *p*< .001, *η_p_^2^*= 0.16; with belief changes being significantly higher for the positive event than negative event. The situation type x depression interaction was significant, *F*(1, 141)= 33.21, *p*< .001, *η_p_^2^*= 0.19. Specifically, while the depressed and control groups did not differ significantly for the positive event, the controls scored significantly lower for the negative event than the depressed group, *t*(145) = 7.53, *p* < .001. The culture x situation type interaction and three-way interaction were non-significant.

**Appraisals**

The situation type main effect was significant, *F*(1, 140)= 12.95 *p*< .001, *η_p_^2^*= 0.09; with agency appraisals being significantly higher for the positive event than negative event. The situation x depression interaction was significant, *F*(1, 140)= 8.81, *p*< .001, *η_p_^2^*= 0.11. While the MDD and control groups did not differ significantly for the positive event, those with MDD had significantly greater agency appraisals for the negative event than the control group, *t*(144)= 2.96, *p*< .01.

**Figure Legends**

*Figure 1.* Mean (±SE) of proportion of correct responses on the Emotion Recognition Task

Iranian MDD

Iranian Control

Malay MDD

Malay Control

Australian MDD

Australian Control

Table 1

*Group Means (and Standard Deviations) for the Emotion Meaning Task*

|  | Malay | | Iranian | | Australian | |
| --- | --- | --- | --- | --- | --- | --- |
|  | MDD (*n* =37) | Control (*n* = 37) | MDD (*n* = 15) | Control (*n* = 15) | MDD (*n* = 19) | Control (*n* = 23) |
| Emotion Meaning  Positive Event | |  |  |  |  |  |
| Social Worth | 11.87 (4.75) | 13.91 (2.89) | 10.80 (3.21) | 12.87 (4.04) | 10.89 (10.05) | 10.85 (4.38) |
| Appraisals | 17.39 (3.59) | 18.20 (3.16) | 16.86 (3.08) | 16.93 (3.99) | 16.04 (3.90) | 16.04 (3.11) |
| Sources | 19.47 (6.31) | 20.70 (4.14) | 15.06 (6.03) | 21.73 (4.88) | 21.28 (4.51) | 22.43 (4.33) |
| Shared | 5.27 (2.93) | 7.08 (1.62) | 5.71 (2.39) | 6.93 (1.83) | 3.11 (1.82) | 2.52 (2.47) |
| Beliefs | 26.51 (6.91) | 28.73 (5.18) | 21.75 (7.63) | 25.47 (5.45) | 26.21 (7.41) | 22.30 (9.09) |
| Emotion Meaning  Negative Event | |  |  |  |  |  |
| Social Worth | 9.96 (4.96) | 6.49 (3.85) | 6.83 (4.11) | 7.20 (4.76) | 7.25 (4.13) | 4.82 (4.07) |
| Appraisals | 17.49 (3.66) | 16.34 (3.50) | 15.92 (3.71) | 11.83 (4.64) | 16.07 (3.43) | 14.55 (3.36) |
| Sources | 16.44 (6.39) | 17.65 (5.63) | 15.94 (7.08) | 17.20 (8.27) | 21.28 (6.00) | 22.91 (3.20) |
| Shared | 5.92 (2.71) | 7.14 (1.90) | 5.59 (2.37) | 7.60 (.91) | 2.53 (2.67) | 1.78 (1.76) |
| Beliefs | 30.03 (7.40) | 17.57 (8.93) | 19.94 (7.89) | 17.20 (8.20) | 25.95 (8.40) | 13.57 (6.29) |

***Note:*** BDI-II = Beck Depression Inventory-II; MDD = major depressive disorder. Sources = sources of appraisals; shared = shared emotion; beliefs = belief changes.

5
